# A Head and Neck Cancer Tumor Response-Specific Gene Signature for Cisplatin, 5-Fluorouracil Induction Chemotherapy Fails with Added Taxanes

**DOI:** 10.1371/journal.pone.0047170

**Published:** 2012-10-09

**Authors:** Céline Tomkiewicz, Stéphane Hans, Marie Hélène Mucchielli, Nicolas Agier, Hervé Delacroix, Laetitia Marisa, Daniel Brasnu, Lawrence P. Aggerbeck, Cécile Badoual, Robert Barouki, Martine Aggerbeck

**Affiliations:** 1 INSERM, UMR-S747, Centre Universitaire des Saints Pères, Paris, France; 2 Université Paris Descartes, Sorbonne Paris Cité, Paris, France; 3 Assistance Publique-Hôpitaux de Paris, Service d’otorhinolaryngologie et de chirurgie cervico-faciale, Hôpital Européen Georges Pompidou, Paris, France; 4 CNRS, Centre de Génétique Moléculaire, UPR2167, Gif-sur-Yvette, France; 5 Gif/Orsay DNA Microarray Platform, Gif-sur-Yvette, France; 6 Université Pierre et Marie Curie (UMPC Sorbonne universités), Paris, France; 7 Université Paris-Sud 11, Centre d’Orsay, Orsay, France; 8 Assistance Publique-Hôpitaux de Paris, Service d’anatomo-pathologie, Hôpital Européen Georges Pompidou, Paris, France; 9 INSERM U970, PARCC (Paris Cardiovascular Research Center), Université Paris Descartes, Paris, France; 10 Assistance Publique-Hôpitaux de Paris, Hôpital Necker-Enfants Malades, Paris, France; Virginia Commonwealth University, United States of America

## Abstract

**Background:**

It is a major clinical challenge to predict which patients, with advanced stage head and neck squamous cell carcinoma, will not exhibit a reduction in tumor size following induction chemotherapy in order to avoid toxic effects of ineffective chemotherapy and delays for instituting other therapeutic options. Further, it is of interest to know to what extent a gene signature, which identifies patients with tumors that will not respond to a particular induction chemotherapy, is applicable when additional chemotherapeutic agents are added to the regimen.

**Methodology/Principal Findings:**

To identify genes that predict tumor resistance to induction with cisplatin/5-fluorouracil (PF) or PF and a taxane, we analyzed patient tumor biopsies with whole genome microarrays and quantitative reverse transcriptase-PCR (TLDA) cards. A leave one out cross-validation procedure allowed evaluation of the prediction tool. A ten-gene microarray signature correctly classified 12/13 responders and 7/10 non-responders to PF (92% specificity, 82.6% accuracy). TLDA analysis (using the same classifier) of the patients correctly classified 12/12 responders and 8/10 non-responders (100% specificity, 90.9% accuracy). Further, TLDA analysis correctly predicted the response of 5 new patients and, overall, 12/12 responders and 13/15 non-responders (100% specificity, 92.6% accuracy). The protein products of the genes constituting the signature physically associate with 27 other proteins, involved in regulating gene expression, constituting an interaction network. In contrast, TLDA-based prediction (with the same gene signature) of responses to induction with PF and either of two taxanes was poor (0% specificity, 25% accuracy and 33.3% specificity, 25% accuracy).

**Conclusions/Significance:**

Successful transfer of the microarray-based gene signature to an independent, PCR-based technology suggests that TLDA-based signatures could be a useful hospital-based technology for determining therapeutic options. Although highly specific for tumor responses to PF induction, the gene signature is unsuccessful when taxanes are added. The results illustrate the subtlety in developing “personalized medicine”.

## Introduction

Head and neck squamous cell carcinoma (HNSCC) is the sixth most frequent cancer worldwide [1]. In France, over 20,000 new cases and about 6,000 deaths were reported in 2003 and the five-year survival rate is still low (50%). Treatment strategies for advanced head and neck cancer have changed over the last 30 years. Strategies today, particularly for laryngeal, oro- and hypopharyngeal cancer, are focused on surgical and non surgical procedures that preserve a functional organ. [2].

Historically, two clinical studies, from the Department of Veterans Affairs (DVA) Laryngeal Cancer Study Group [3] and the Radiation Therapy Oncology Group (RTOG) 91–11 [4], have influenced the management of advanced laryngeal cancer. The DVA study [3] was the first to promote the organ preservation strategy and to prove that patient survival after neoadjuvant or induction chemotherapy (cisplatin and 5-fluoruracil) followed by radiation therapy was almost identical to that after total laryngectomy and postoperative radiotherapy.

However, the use and benefit of chemotherapy remained the subject of debate following a meta-analysis, in 2000, of 63 trials between 1965 and 1993, which showed that chemotherapy on non-metastatic head and neck squamous cell carcinoma in 10,741 patients with carcinoma of the oropharynx, oral cavity, larynx or hypopharynx gave only a small significant survival benefit for concomitant chemoradiation therapy [5].

In 2007, an Eastern Cooperative Oncology Group phase II multicenter study [6] reported a high organ-preservation rate with taxane-based chemotherapy for oropharyngeal cancer but not for laryngeal cancer. Two clinical studies [7], [8] were published in 2007 which compared a more intensive induction chemotherapy regimen; docetaxel was added to the conventional cisplatin/5-fluoruracil regimen. Sequential therapy with induction docetaxel, cisplatin and 5-fluoruracil (TPF) significantly improved survival and progression-free survival versus cisplatin and 5-fluoruracil (PF) in locally advanced laryngeal, oro- and hypopharyngeal cancer [7] suggesting the use of sequential TPF followed by carboplatin chemo-radiotherapy as a treatment option for organ preservation and to improve survival in locally advanced laryngeal, oro- and hypopharyngeal cancer. The European TAX 323 study group [8] compared TPF with PF as induction chemotherapy in patients with locoregionally advanced, unresectable disease and showed that the median progression-free survival was 11.0 months in the TPF group in comparison to 8.2 months in the PF group. Although the addition of the taxane docetaxol to cisplatin/5-fluorouracil induction chemotherapy improves the clinical response and survival as compared to cisplatin/5-fluorouracil alone or cisplatin/5-fluorouracil combined with radiotherapy, it may have a higher incidence of adverse haematological events (neutropenia and related complications) [9]. Nevertheless, about 30% of patients show either no improvement or worsening of their condition after induction. It is, thus, a major clinical challenge to predict which patients will not benefit from induction chemotherapy i) to avoid toxic effects of ineffective chemotherapy; ii) to avoid delays for other therapeutic options and iii) to minimize the cost of treatment.

Previous investigations of the response to induction chemotherapy in HNSCC showed that differences in the genotypes of various enzymes are associated with variations in the response to cisplatin-based induction chemotherapy [10]. Cyclin A expression in HNSCC predicted a better response to cisplatin/5-fluorouracil chemotherapy [11]. Also, better survival indices for patients were found when the expression of laminin and syndecan-1 changed in response to chemotherapy [12]. However, all these studies focus on only one or a few genes. Microarray-based gene expression profiling of head and neck cancers has been used mainly for the classification of tumors or to predict distant metastasis or outcome [13]–[15]. Although gene-expression analyses have assessed the response to preoperative chemo-radiation [16] or induction chemotherapy with 5-fluorouracil, cisplatin and adriamycin [17] in head and neck cancer, no genome-wide microarray study has addressed cisplatin/5-fluorouracil induction therapy. In this study using genome-wide microarray analysis, we sought to identify genes that were predictive of a tumor response to induction chemotherapy with cisplatin/5-fluorouracil. We further sought to determine to what extent this gene signature was applicable when additional chemotherapeutic agents (cisplatin/5-fluorouracil and a taxane-docetaxel or paclitaxel) were added to the treatment regimen.

**Table 1:** Patient Characteristics.

Patient	Age*	Cigarettesper day	Alcohol per day (L/day)	Hemoglobin (g/dL)	Therapy	Class^+^	Study	Status^†^
23	63	40	>1	12.1	PF	CCR	MA	A
40	45	50	<1	15.1	PF	CCR	MA/TLDA	A
57	44	40	>2	14.4	PF	CCR	MA/TLDA	A
109	60	40	1	13.3	PF	CCR	MA/TLDA	A
110	56	45	<1	12.8	PF	CCR	MA/TLDA	A
121	44	45	>2	12.8	PF	CCR	MA/TLDA	D
130	44	45	3	12.1	PF	CCR	MA/TLDA	D
156	56	40	>3	12.7	PF	CCR	MA/TLDA	D
157	71	0	0	11.1	PF	CCR	MA/TLDA	A
164	60	20	0	11.4	PF	CCR	MA/TLDA	A
169	58	65	1	12.7	PF	CCR	MA/TLDA	A
195	47	30	1	12.1	PF	CCR	MA/TLDA	A
219	52	40	1	12.1	PF	CCR	MA/TLDA	A
48	55	55	>2	11.1	PF	NR	MA/TLDA	D
140	50	10	1	12.1	PF	NR	MA/TLDA	A
143	62	20	1	13.1	PF	NR	MA/TLDA	A
159	58	10	1	12.1	PF	NR	MA/TLDA	A
198	63	20	1	12.1	PF	NR	MA/TLDA	D
210	59	10	2	11.4	PF	NR	MA/TLDA	A
223	65	40	2	11.9	PF	NR	MA/TLDA	D
246	66	30	2	12.9	PF	NR	MA/TLDA	D
302	69	40	>2	12.1	PF	NR	MA/TLDA	D
303	58	20	>2	12.9	PF	NR	MA/TLDA	A
27	50	20	>2	12.8	PF	NR	TLDA	D
39	43	15	1	12.1	PF	NR	TLDA	A
76	48	0	<1	14.1	PF	NR	TLDA	D
318	62	60	>2	11.1	PF	NR	TLDA	D
329	49	40	>1	12.2	PF	NR	TLDA	A
310	58	90	>2	11.2	T1PF	CCR	TLDA	A
323	45	10	<1	12.5	T1PF	CCR	TLDA	A
353	52	25	>1	11.5	T1PF	CCR	TLDA	D
374	62	35	>1	12	T1PF	CCR	TLDA	D
138	54	20	1	11.2	T1PF	NR	TLDA	A
162	58	25	>2	10.8	T1PF	NR	TLDA	D
174	47	30	1	11.5	T1PF	NR	TLDA	D
350	69	30	1	11.6	T1PF	NR	TLDA	D
387	53	20	1	11.5	T2PF	CCR	TLDA	A
395	61	10	1	12.1	T2PF	CCR	TLDA	A
413	64	10	1	11.5	T2PF	CCR	TLDA	A
416	62	20	1	12.1	T2PF	CCR	TLDA	A
417	65	15	2	12.1	T2PF	CCR	TLDA	A
419	51	10	0	12.1	T2PF	CCR	TLDA	A
213	50	40	3	12	T2PF	NR	TLDA	D
427	55	20	1	11.5	T2PF	NR	TLDA	A

Abbreviations: *Age at diagnosis; ^+^Response to induction therapy, CCR, complete clinical responder; NR, non-responder; **^†^** Alive (A) or Deceased (D) in 2009 ; MA, microarray; TLDA, Taqman low density array card; PF, Cisplatin/5FU; T1PF, Cisplatin/5FU/Paclitaxel; T2PF, Cisplatin/5FU /Docetaxel.

**Table 2:** Characteristics of Patients (Microarray Study).

	Response to Chemotherapy											
	Nonresponders				Responders				All Patients			
Characteristics	No. of Patients			%	No. of Patients		%	*P*	No. of Patients			%
**Sex (Male)**	10			43	13		57	0.74	23			100
**Age (Years)**												
Mean	60.1+/−5.6				53.8+/−8.7			0.06	56.7+/−8.1			
Median	61				54				57			
Range	50–69				44–71				44–71			
**Tumor localization**	Oropharynx											
**Histology**	Squamous cell carcinoma											
**Metastases**	None											
**Smoking (Cig./day)**								0.05				
0	0		0		1	8			1	4		
1–20	6		60		1	8			7	30		
21–40	3		30		6	46			9	39		
41–60	1		10		4	31			5	22		
61–80	0		0		1	8			1	4		
**Alcohol (Liters/day)** **								0.45				
0	0		0		2	15			2	9		
≤1	4		40		6	46			10	43		
>1≤2	3		30		1	8			4	17		
>2≤3	3		30		3	23			6	26		
>3	0		0		1	8			1	4		
**Hemoglobin**	12.2+/−0.6				12.7+/−1.1			0.19	12.5+/−1.0			
**HPV Status**								0.03*				
**HPV (+)**	3	30			10	83			13		59	
**HPV (−)**	7	70			2	17			9		41	

* The probability was calculated with the Fisher Exact Test.

** Alcohol was consumed predominantly in the form of red wine.

Note that the HPV status could not be ascertained for one individual of the Responder group.

**Table 3:** Characteristics of Patients (TLDA Study).

	Response to Chemotherapy				Response to Chemotherapy				Response to Chemotherapy						
	PF				T1PF				T2PF						
	Non- responders		Responders		Non-responders		Responders		Non- responders		Responders			All Patients	
	(n = 15)		(n = 12)		(n = 4)		(n = 4)		(n = 2)		(n = 6)			(n = 43)	
Characteristics	No. of Patients	%	No. of Patients	%	No. of Patients	%	No. of Patients	%	No. of Patients	%	No. of Patients		%	No. of Patients	%
**Sex (Male)**	15	56	12	44	4	50	4	50	2	25	6		75	43	100
**Age (Years)**															
Mean	57.1+/−7.7		53.1+/−8.7		57.0+/−9.2		54.2+/−7.4		52.5		59.3+/−5.9			55.8+/−7.7	
Median	58		54		56		55		53		62			56	
Range	43–69		44–71		47–69		45–62		50–55		51–65			44–71	
**Cigarettes/day**															
0	1	7	1	8	0	0	0	0	0	0	0		0	2	5
1–20	8	53	1	8	1	25	1	25	1	50	6		100	18	42
21–40	4	27	5	42	3	75	2	50	1	50	0		0	15	35
41–60	2	13	4	33	0	0	0	0	0	0	0		0	6	14
61–80	0	0	1	8	0	0	1	25	0	0	0		0	2	5
**Alcohol (L/day)**															
0	0	0	2	17	0	0	0	0	0	0	1		17	3	7
≤1	6	40	6	50	3	75	1	25	1	50	4		67	21	49
>1≤2	4	27	0	0	0	0	2	50	0	0	1		17	7	16
>2≤3	5	33	3	25	1	25	1	25	1	50	0		0	11	26
>3	0	0	1	8	0	0	0	0	0	0	0		0	1	2
**Hemoglobin**	12.3+/−0.8		12.7.+/−1.1		11.3+/−0.4		11.8+/−0.6		11.8+/−0.4			11.9+/−0.3		12.2+/−0.9	

## Methods

### Patients and Induction Chemotherapy

Between 2002 and 2007, patients with histologically proven oropharyngeal carcinoma without a previous history of cancer or multiple tumor locations and lacking contraindication for cisplatin-based chemotherapy were enrolled in the study at the Georges Pompidou European Hospital (HEGP), Paris. The patients, all of whom had advanced (stage 3 or 4) cancers, received either PF (100 mg/m^2^ cisplatin Day (D) 1 and 1 g/m^2^ 5-FU D1–D5) or TPF (either carboplatine (paraplatine)-AUC5 D1 (where AUC is area under the curve, a formula that allows calculation of the dose by adapting it to the value of the creatinine clearance) and 175 mg/m^2^ paclitaxel D1 (T1PF) or PF plus docetaxel: 75 mg/m^2^ cisplatin D1, 75 mg/m^2^ docetaxel D1, and 750 mg/m^2^ 5-FU as a continuous perfusion D1–D5 (T2PF) induction chemotherapy. An average of 3 courses (range 2–5) was given with 14–21 day intervals between the courses and the dosages were adjusted to take into account individual tolerance and response. The patients were evaluated by the Head and Neck Tumor Board of the HEGP (composed of head and neck specialists, radiologists and pathologists). We studied the patients in two of the four groups in the ECOG classification [18], in order to have two groups that were the most “clinically” different. The response was defined by both clinical and radiological examination; complete clinical responders (CCR) showed more than 90% decrease of the tumor size whereas the non-responder group (NR) showed less than 50% decrease in tumor size or progression of the disease.

**Table 4:** Gene Signature That Predicts Response to PF.

Name	ID	MicroArraysFold Change(NR/R)	MicroArraysp-value(NR/R)	TLDA Fold Change (NR/R)	TLDA p-value (NR/R)	Gene Functions		
**ODZ2**	AL137500	3.45	2E-5	6.04	0.0042		Signal transduction.	
**DLL1**	NM_005618	1.82	6 E-4	2.65	0.0025		Signaling, differentiation, development, Notch signaling pathway, cell communication, cell fate determination, compartment pattern formation, determination of left/right symmetry, negative regulation of cell differentiation, regulation of cell adhesion, somite specification.	
**PLSCR4**	NM_020353	−1.35	0.004	−3.23	0.0014		Phospholipid scrambling, may mediate accelerated ATP-independent bidirectional transbilayer migration of phospholipids upon binding calcium ions that results in a loss of phospholipid asymmetry in the plasma membrane. May play a central role in the recognition of apoptotic and injured cells by the reticuloendothelial system.	
**ZNF462**	NM_021224	1.41	0.016	1.39	0.1996		Negative regulation of DNA binding, Positive regulation of transcription, zinc ion binding may be involved in transcriptional regulation.	
**TXNDC9 (APACD)**	NM_005783	1.44	0.019	1.06	0.720		Cell redox homeostasis, protein binding significantly diminishes the chaperonin TCP1 complex ATPase activity, thus negatively impacts protein folding, including that of actin or tubulin.	
**TCP1**	NM_030752	1.33	0.028	1.06	0.674		Molecular chaperone, protein folding, assists the folding of proteins upon ATP hydrolysis, known to play a role, in vitro, in the folding of actin and tubulin complex assembly.	
**SSB**	NM_003142	1.37	0.246	1.06	0.675		RNA metabolism, binding and processing, binds to the 3′ poly(u) terminii of nascent RNA polymerase III transcripts, protecting them from exonuclease digestion and facilitating their folding and maturation.	
**RPL10**	BM981501	−1.39	0.022	−1.52	0.156		Possible tumor suppressor, translation regulation.	
**MORF4L1**	NM_006791	1.27	0.151	−1.32	0.340		Histone acetylation, deacetylation, DNA recombination, repair (homologous recombination), chromatin modification, regulation of growth, involved in transcriptional activation of select genes principally by acetylation of nucleosomal histones which may both alter nucleosome - DNA interactions and promote interaction of the modified histones with other proteins which positively regulate transcription. May be involved in the activation of transcriptional programs associated with oncogene and proto-oncogene mediated growth induction, tumor suppressor mediated growth arrest and replicative senescence, apoptosis, and DNA repair, also component of the msin3a complex which acts to repress transcription by deacetylation of nucleosomal histones.	
**DNAJA1 (HSP40)**	NM_001539	1.11	0.075	−1.04	0.839		Protein folding, degradation, protein binding, response to unfolded protein, co-chaperone of HSP70, seems to play a role in protein import into mitochondria. DNA damage response, detection of DNA damage.	

**Table 5:** Statistical Summary for 10 Gene Predictor.

True condition = Non-responder					
Induction therapy	PF			T1PF	T2PF
**Gene Expression Technology**	MA	TLDA*	TLDA**	TLDA	TLDA
**Number of Patients**	23	22	27	8	8
**Number of Predicted Non Responders/** **Non Responders**	7/10	8/10	13/15	2/4	0/2
**Number of Predicted Responders/** **Responders**	12/13	12/12	12/12	0/4	2/6
**Sensitivity/True Positive Rate/Power**	70%	80%	86.7%	50%	0%
**Specificity/True Negative Rate**	92%	100.0%	100.0%	0%	33.3%
**Positive Predictive Value**	88%	100.0%	100.0%	33%	0%
**Negative Predictive Value**	80%	86%	85.7%	0%	50.0%
**False Positive Rate (α)**	7.7%	0.0%	0.0%	100%	66.7%
**False Negative Rate (β)**	30%	20%	13.3%	50%	100%
**Accuracy (ACC)**	82.6%	90.9%	92.6%	25%	25%
**False Discovery Rate (FDR)**	12.5%	0%	0%	67%	100%

* The samples in this group contained all but one of the samples analyzed by MA. **Samples in this group contained the 5 additional samples analyzed by TLDA that were not analyzed by MA.

**Figure 1:**
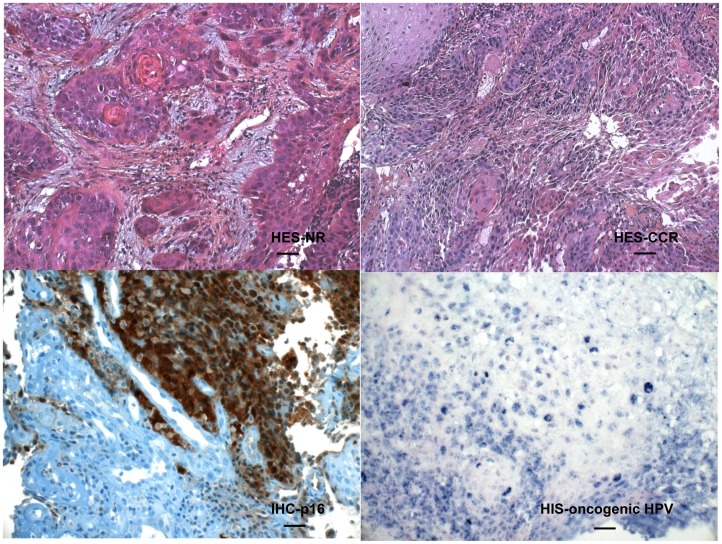
Representative HES, p16 and HPV staining of biopsies from NR and CCR individuals. HES-NR and HES CCR correspond to hematoxylin-eosin-safran staining of representative non-responder and complete clinical responder individuals, respectively, and IHC-p16 and HIS-oncogenic HPV correspond to representative positive immunohistochemistry for the p16 antigen (brown staining) and hybridization *in situ* for oncogenic HPV DNA (blue punctate nuclear staining), respectively. The bar represents 40 microns in the upper panels and 20 microns in the lower panels.

### Ethics

The study was authorized by the ethics committee (CCPPRB Paris-Broussais-HEGP N°2002-035) and obeyed French biomedical research legislation. Informed written consent was obtained from all the participants.

### Sample Collection, Histology and Virology

For diagnostic purposes, tumor samples were obtained during endoscopy under general anesthesia before any treatment. Each tumor biopsy was immediately put into RNAlater (Ambion) to avoid degradation of RNA. The biopsy was cut into 2 pieces, one for pathological examination and the other one was immediately frozen at −80°C until further processing for RNA extraction. Formalin-fixed, paraffin-embedded tissue blocks were used to prepare slides for 1) hematoxylin-eosin-safran (HES) staining for the evaluation of tumor status and percent of tumor cells in the biopsy, 2) immunohistochemical staining for the p16 antigen and 3) *in situ* hybridization using the INFORM HPV (Human Papilloma Virus) III Family 16 Probe (B) kit to detect the main HPV DNA oncogenic types 16, 18, 31, 33, 35, 39, 45, 51, 52, 56, 58, 59, 66, 68 (Roche Diagnostics, France). Staining was performed using the BenchMark Clyde ULTRA Ventana Roche apparatus. The manufacturers’ protocols were used. The HPV status could not be ascertained for one CCR individual of the PF treated group. Histological analyses established that all the tumors were squamous cell carcinomas (Figure 1 shows representative HES staining of biopsies from NR and CCR individuals). The NR and CCR groups exhibited similar overall extents of tumor differentiation and percent of tumor cellularity.

**Figure 2:**
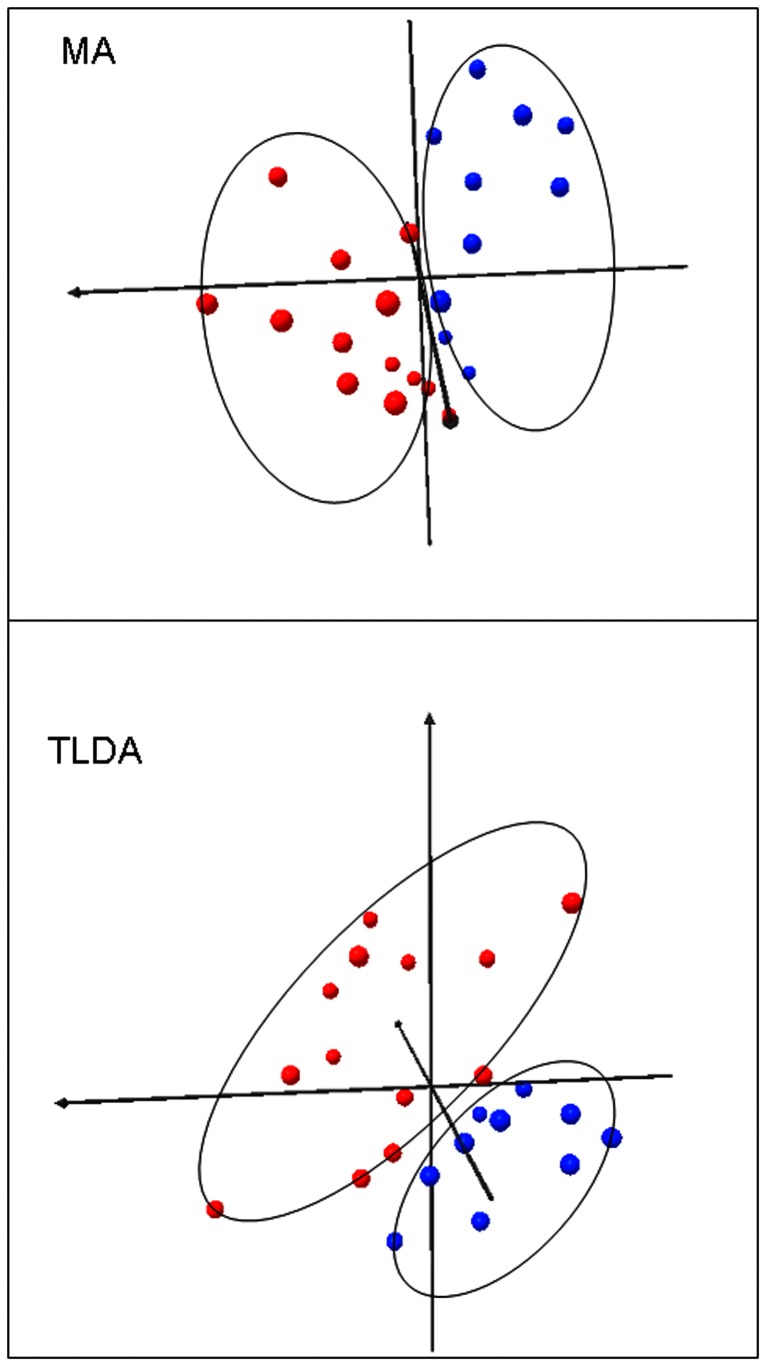
Multidimensional scaling in three dimensions (using Euclidean distances) of the microarray (MA) and TLDA (TLDA) gene expression data. The 10-gene classifier clearly separates the members of the NR (blue spheres) and CCR (red spheres) groups. 82–83 per cent of the variability is contained in these first three dimensions.

### Preparation and Assessment of RNA Quality

“Tripure isolation reagent” (Roche) was used to extract RNA from the biopsy. Briefly, the biopsy (less than 100 mg) was homogenized in 1 mL Tripure with 3 sterile tungsten beads in a Retsch MM20 Mixer Mill (3 min grinding at speed 29, 3 times). After addition of two hundred µL of chloroform, the tube was shaken vigorously at room temperature for at least 5 min and allowed to sit for another 5 min before centrifugation (15 min, 12,500 rpm, 4°C). The aqueous upper layer, containing RNA was collected. After the addition of 500 µL isopropanol and incubation at −20°C for 10 min, the RNA was pelleted by centrifugation (15 min, 12,500 rpm, 4°C). The pellet was washed with 1 mL of 75% ethanol, centrifuged as described above and dried at room temperature for 10 min. Total RNA were re-suspended into 40 µL of RNase-free water. RNA was further purified with the RNase-free DNase Set (Qiagen) and RNeasy minielute cleanup (Qiagen), according to the manufacturer’s protocol. RNAs were quantified using a Nanodrop ND-1000 spectrophotometer and their quality was assessed by electrophoresis using a BioAnalyzer (Agilent).

### Labeling and Purification of Probes

For the microarray studies, a total of 23 samples and a universal RNA reference, comprising RNA from 10 different human tissues (Clontech), were used to synthesize labelled cRNA. Briefly, 200–300 ng total RNA extracted from the biopsies or from the RNA reference were labelled using cyanine3-CTP or cyanine5-CTP and the “Low RNA Input Fluorescent Linear Amplification Kit” (Agilent), according to the manufacturer’s instructions. The fluorescent probes were purified (Purification RNeasy Mini Kit, Qiagen) and the concentration of the probes and the level of incorporation of the dyes into the probes was measured (Nanodrop D-1000 spectrophotometer). Agarose electrophoresis of the labelled probes (1.2% agarose gel in 0.5×TBE) performed on a microscope slide together with DNA markers (100 bp, Biolabs) was performed and the fluorescence of the gel was assessed in a Genetac IV scanner (Perkin-Elmer). Both the intensity of the signal and the profile of the samples indicated the quality of the labelling.

### Hybridization and Washing of Microarrays

The probes (1 µg each of a biopsy sample and of the reference, direct design) were then hybridized to a pan-genomic human 44K microarray (Agilent) containing 41,000 probes, corresponding to genes or expressed sequence tags, using the Agilent 60-mer oligo microarray processing protocol (Agilent). Hybridization was performed for 17 hours at 60°C with rotation in a hybridization oven (Agilent) as described by the manufacturer. The microarrays were washed as described in the manufacturer’s protocol and dried immediately in a speed-vac centrifuge for 2 minutes.

### Array Scanning and Image Processing

The microarrays were scanned using an Axon 4000B scanner (Molecular Devices, Sunnyvale, CA, USA) at 5 µm resolution. PMT voltages were automatically adjusted to balance the distributions of the red and green intensities and to optimize the dynamics of image quantification. The rate of saturated pixels was limited to 0.01%. The resulting 16 bit images were analyzed and the quantification of the signals on the arrays was performed using GenePix Pro 6.0 software (Axon Instruments, Union City, CA). Segmentation was computed with the “adaptive circle” method of processing. Backgrounds were not subtracted and not found, saturated and bad spots were discarded. Intra-array normalization (excluding control spots, 2,615 probes) was performed using the loess method with a 0.5 span parameter.

### Statistical Analysis

Genes differentially expressed between CCR and NR in the microarray study were identified by a mutivariable permutation test controlling the false discovery rate (a moderated t-test with adjustment of p-values [19] using the MAnGO software [20]. Genes were selected as significantly differentially expressed when the p-value was less than 0.025, the mean fold change (the fold change between the means of normalized signals of the 13 complete clinical responders and of the 10 non responders) greater than 1.3 in absolute value and the mean intensity (log_2_(Cy5*Cy3)/2) greater than 7. Each gene has at least one value between the two averages of log_2_(NR/ref) and log_2_(CCR/ref) greater than 0.4 in absolute value and at least one value between the two standard deviations of log_2_(NR/ref) and log_2_(CCR/ref) less than 0.5. Moreover, there is minimal recovery between CCR and NR (|mean(log_2_(NR/ref))–mean(log_2_(CCR/ref))|–[SD(log_2_(NR/ref))+SD(log_2_(CCR/ref))])>−0.55.

A classifier, to predict the response to chemotherapy of future patients from the genes selected as differentially expressed, was devised with a supervised prediction method (Support Vector Machines with linear kernel, SVM) [21], [22] using BRB Array Tools Version 4.1.0 developed by Dr. Richard Simon and BRB Array Tools Development Team [23]. The classifier was evaluated [23], [24] by using a leave-one-out cross-validation (LOOCV) procedure. Multidimensional scaling (Euclidean distances) was performed with the SMACOF algorithm [25] and the Kruskal stress formula [26]. The cross-validation step also allows the designation, among the genes selected, of those that give the best scores of prediction. It is these genes whose signature will be used to determine the response to chemotherapy. ROC curve analysis was performed with the Sigma Plot 11 data analysis and graphing software package. Comparison of the clinical-biological characteristics of the NR and CCR groups was made using the t-test or Mann-Whitney statistic when appropriate.

### Reverse Transcription of RNA for PCR Analysis

cDNA was prepared from 200 ng RNA using the High Capacity cDNA Archive kit (Applied Biosystems). In order to compare the level of mRNA in the various biopsies, the same universal RNA reference was used as that in the microarrays.

### Taqman Low Density Array (TLDA)-quantitative RT-PCR

Forty-three biopsies from patients treated with PF (22 of the 23 biopsies that were analyzed with microarrays (there was insufficient RNA from one of the original patients to be used in the TLDA study), plus five additional biopsies from patients who received the same PF induction therapy), 8 biopsies from patients treated with T1PF and 8 biopsies from patients treated with T2PF were used in the TLDA study. Quantitative RT-PCR was performed with Taqman low density array cards. Pre-designed Taqman probe and primer sets for the classifier target genes (based upon the microarray results) were factory-loaded into the 384 well array. The array format was customized on line with 2 replicates per classifier target gene. The cDNA samples were analyzed using an ABI Prism 7900HT apparatus according to the manufacturer’s instructions. Briefly, each cDNA sample (100 ng in 25 µL) was combined with 25 µL water and added to an equal volume of 2X Taqman Universal PCR Master Mix (Applied Biosystems). The mixtures (100 µL) were injected into a loading port of the TLDA. For each TLDA, cDNA from the Clontech universal reference was used in parallel with 7 of the samples to evaluate the reproducibility of the experiments. The array was centrifuged twice to distribute the samples into the wells. The card was sealed and the amplification was performed as follows: 2 min at 50°C (activation of uracil-DNA glycosylase), 10 min at 94.5°C (activation), 40 cycles of denaturation at 97°C for 30 sec and 1 min at 59.7°C (annealing and extension). Gene expression values were calculated based on the threshold cycle (Ct) method, which uses the formula 2^−ΔΔCt^ to calculate the expression of target genes normalized to a calibrator (Clontech universal reference). Briefly, the C_t_ data for all the target genes and the GAPDH gene in each sample was used to create the ΔC_t_. Thereafter, ΔΔC_t_ values were calculated by substracting the ΔC_t_ of the calibrator from the ΔC_t_ of the sample. The relative quantities (RQ) were determined using the equation RQ = 2^−ΔΔCt^. The sample values were expressed relative to the calibrator sample.

### Analysis of Protein-Protein Interactions

Manual interrogation of databases (PSIQUIC View: http://www.ebi.ac.uk/Tools/webservices/psicquic/view/main.html; MINT: http://www.mint.bio.uniroma2.it/mint/; InnateDB: http://ww.innatedb.com; IntAct: http://www.ebi.ac.uk/Databases; BioGrid: http://www.thebiogrid.org and BioLink Explorer: http://www.diatomsoftware.com/biolink/) was performed to identify protein interactors which for which there was experimental evidence for direct physical association with the proteins encoded by the genes of the signature. Then, experimental evidence was sought in the same databases for evidence of direct association between the interactors. The resulting network was visualized in Cytoscape: http://www.cytoscape.org/.

## Results

### Clinical Characteristics and Response to Induction Chemotherapy

A total of 44 male patients were enrolled (Table 1). Women were excluded since the etiology for HNSCC may be different from that in men. Among the clinical characteristics (Tables 1 and 2) of the patients treated with only PF in the microarray study, only the HPV status and cigarettes smoked per day differed significantly (p = 0.03 and 0.05, respectively) between the two groups (the CCR group exhibited more HPV positivity and smoked more). Among the patients in the TLDA study, there were no significant differences in the clinical characteristics of the NR and CCR. NR and CCR were evenly divided among the 8 patients treated with PF and paclitaxel (T1PF) whereas 6 of the 8 patients treated with PF and docetaxel (T2PF) were CCR (Table 3). Discriminant analysis of the ensemble of the patients using the age, tumor stage, consumption of cigarettes and alcohol, and levels of haemoglobin did not predict the response to chemotherapy (predictive score less than 70%).

### A Gene Signature for Response to Cisplatin/5-fluorouracil Induction

Using the microarray results from the biopsies of the 23 patients treated with PF (GEO database: GSE32877), a ten-gene signature (Table 4) for response to therapy was derived. Receiver operating characteristic curves (ROC curves) of each of the ten genes showed their significance for classifying non-response to chemotherapy (Figures S1 and S2). This classifier correctly classified twelve of thirteen CCR and 7 of the 10 NR (92% specificity, 70% sensitivity, overall accuracy of 82.6%, Table 5). The classifier was used, then, to design Taqman Low Density Arrays (TLDA), a technology more readily adapted to hospital-based analyses than the microarray technology.

TLDA containing the 10-gene microarray-derived classifier were used to perform quantitative RT-PCR on a total of 43 biopsies (Tables 1 and 3 and Table S1). Of the 22 patients treated with PF only, that were in the microarray study, 12 of 12 CCR and 8 of 10 NR were correctly classified by TLDA using the classifier (100% specificity, 80% sensitivity and 90.9% overall accuracy and Figure S2). TLDA analysis also correctly predicted the response of all 5 new patients treated with PF and, overall (for the 27 patients treated with PF only), correctly classified 12 of 12 CCR and 13 of 15 NR (100% specificity, 86.7% sensitivity and 92.6% overall accuracy) (Table 5 and Figure S2). Multidimensional scaling (3D) shows that the descriptors of the classifier clearly separate the CCR and NR individuals into two groups based on the microarray (MA) and TLDA data (Figure 2). Although the HPV status of individuals was found to be different between CCR and NR groups (22 patients), addition of the HPV status to the 10 gene signature did not improve the predictions for the TLDA analysis and actually misidentified one of the 5 new patients (the ROC curve is shown in Figure S2). There was no significant difference in HPV status of individuals in the 27 patient CCR and NR groups. Interestingly, experimental evidence was found in several databases for direct physical interaction among the protein products of the genes of the 10 gene signature and other interactor proteins which form a network (Figure S3 and Table S2).

### The Signature is Specific for the Induction Chemotherapy Regimen

To determine its specificity to the drug regimen, we investigated the performance of the classifier in 16 additional patients treated either with paclitaxel or docetaxel plus PF (T1PF and T2PF, respectively, Tables 1 and 3). Using the TLDA results obtained with biopsies from patients treated with either T1PF or T2PF, the classifier poorly predicted the response to chemotherapeutic induction (0% specificity, 50% sensitivity, 25% accuracy and 33.3% specificity, 0% sensitivity, 25% accuracy, respectively, Table 5). For T1PF induction, the classifier identified only 2 of 4 NR correctly and 0 of 4 CCR. Neither of the 2 NR was correctly identified in the case of T2PF induction and only 2 of 6 CCR were identified.

## Discussion

Oropharyngeal squamous cell carcinomas (particularly of the base of the tongue and the tonsil) represent 35–40% of head and neck cancers in France (4000 cases/year) and their incidence has increased in western countries over the last decade. Here, we studied advanced stage cancers of the oropharynx because they are the most frequent thus permitting recruitment of a sufficient number of patients to compare complete clinical responders with non-responders for induction chemotherapy.

Since no association was found between the clinical characteristics and the response to chemotherapy, a gene signature is relevant. This study is the first to employ whole genome transcriptome analysis of tumor biopsies to identify a set of genes predictive specifically for the tumor response to PF induction chemotherapy for HNSCC patients and to transpose that gene signature to a technology (TLDA) that may be suitable for hospital-based use. Determination of this signature was contingent upon the use of a homogeneous subset of male patients who had identical localizations of their cancer and who belonged to one or the other of the two extreme groups of NR and CCR. The microarray-based classifier is highly specific for NR.

We also designed and tested a Taqman Low-Density Array in order i) to compare, using the same patients, the sensitivity, specificity and accuracy of the microarray-based classifier with an independent technology (quantitative RT-PCR) more readily adapted for hospital-based analyses; ii) to evaluate the accuracy of the signature for patients who were not in the microarray study.

The microarray technology-derived classifier was successfully transposed to a PCR-based technology (TLDA cards) and gave similar predictive results. Further, using the TLDA technology, the classifier correctly classified all the new patients. Overall, for the TLDA assay, none of the 12 CCR was misclassified as NR and 13 of 15 NR were correctly identified. These results suggest that a microarray classifier can be transposed to a technology that is more appropriate for hospital-based use and, if further validated, the technology could be potentially useful for specific chemotherapy-related decisions. Viewed from a therapeutic standpoint, since all the predicted non-responders were true non-responders and only 2 of the predicted responders were, in fact, non-responders, this indicates, potentially, that on the one hand, a minimum of individuals would undergo induction therapy from which they will not benefit positively and which will delay alternative therapeutic options and, on the other hand, therapy would not be withheld from individuals who might benefit. However, the signature reported here is appropriate neither for the classification nor the diagnosis of HNSCC or for the prediction of metastases, prognosis or survival and it has few or no genes in common with studies of HNSCC having these goals.

### Functional Significance of the Genes in the Signature

It was not possible, nor was it the objective of this study, to determine whether a gene in the classifier is causal or simply a marker for the non-response. The majority of the genes in the classifier (8/10) were overexpressed in the NR tumors. Three of the up-regulated genes in NR (TCP1alpha, TXNDC9 and DNAJA1) code for proteins that participate in protein folding and cellular assembly and organization. TCP1alpha/CCT1 is part of the chaperonin TCP1 ring complex [27], [28]. In several cancers, TCP1 protein family members are overexpressed [29] and chemoresistance has been linked to increased chaperone protein expression [30] suggesting that rapidly growing tumors need increased machinery for the correct folding of proteins [31]. TXNDC9, also called APACD (ATP binding protein associated with cell differentiation), has been reported to diminish the chaperonin TCP1 complex ATPase activity and, thus, negatively impact protein folding [32]. Moreover, the gene is up-regulated in oxaliplatin resistant ovarian and head and neck carcinoma cell lines [33], in accordance with our findings. DNAJA1 (HSP40), with HSP70 and co-chaperones, constitutes a proteostasis maintenance system. Increased expression of HSPs is found in malignancy and HSP70, with which DNAJA1 associates, may be involved in resistance to chemotherapy [34].

Two other up-regulated genes (DLL1 and ODZ2) code for proteins that are involved in diverse signaling processes. DLL1 (Delta-like 1) is a Notch receptor ligand [35]. Recently, mutations in Notch1,2,3 have been described in head and neck cancers [36] and alterations in the expression of DLL1 have been described in several tumors, including oral squamous cell carcinoma [37] and urinary bladder cancer [38].

Finally, the protein products of three other up-regulated genes (MORF4L1, SSB and ZNF462) participate in nucleic acid binding and transcriptional control. MORF4L1 (Mortality Factor 4) is a member of the MRG (Morf-related genes) family which is important for transcriptional control, DNA damage repair, proliferation and cellular aging. MORF4 is a transcription factor-like protein that induces senescence in immortalized cells [39]. MORF4 is up-regulated in high degree microsatellite instability colorectal carcinoma [40]. The SSB protein product, also known as La protein, is an abundant, essential RNA-binding protein involved in several facets of RNA metabolism and in particular in the stabilization of small RNAs from exonuclease digestion [41]. In various tumors, La contributes to cell proliferation and migration and also to invasion of lymph node-metastasized hypopharyngeal squamous cell carcinoma cells [42]. Finally, the ZNF 462 gene encodes a putative C2-H2 Zn-finger protein presumed to be involved in DNA binding and the regulation of transcription.

PLSCR4 or scramblase 4, the product of one of two down-regulated genes in the NR belongs to a family of proteins of which PLSCR1 [43], in particular, has been implicated in gene regulation, immuno-activation and cell proliferation/apoptosis [44]. PLSCR1 can suppress tumorogenesis by inducing cell differentiation in ovarian and leukemic cells [45] and its expression has been proposed as a new positive prognostic marker for acute myelogenous leukemia [43]. The other down-regulated gene is RPL10 (also called QM and DNASE1L1). *In vitro* studies have suggested that the protein product of RPL10 may be a tumor suppressor [46].

### Limitations

Finally, the results illustrate some caveats related to the development of “personalized medicine”. Although major advances using large scale, high throughput studies continue to be made to link drug sensitivity to cancer genetics and gene expression with the goal of identifying potential genomic markers of drug sensitivity in cancer cells [47]–[49], a major problem continues to be the heterogeneity in the responses of individuals affected with the same disease to a given therapeutic agent. This heterogeneity is underscored by the present study, even though it contains a modest number of patients.

Having identified a gene signature that allows the efficient classification of patients into responder and non-responder groups when treated with a particular induction chemotherapeutic regimen, it might have been expected that the same gene signature also would identify the individuals with tumors that are sensitive to treatment with the same regimen to which a taxane is added. This is not the case.

The gene signature appears to be very specific for the induction therapy used. Indeed, using the TLDA assay, the classifier was incapable of discriminating among 16 new CCR and NR patients treated with T1PF or T2PF induction chemotherapy (PF to which a taxane had been added) although still correctly classifying all 5 new patients treated with PF induction only. Thus, the gene classifier does not identify individuals who will respond, in general, to chemotherapy but rather identifies individuals who will respond to a specific chemotherapeutic protocol. The unique genetic constitution and environment of each individual may contribute to his/her response to therapy thus explaining, in part, the different responses to the same drug regimen as well as to different regimens. Similarly, different molecular mechanisms could be involved in the production of a given disease phenotype which respond differently to therapeutic agents believed to affect the same pathways. Further complicating the goal of personalizing medicine is the often disconnected manner in which continuing rapid advances in technology (“omics” of all types) and evolution of therapies (for example during the course of this study taxanes were found to be a useful addition to PF induction therapy) may occur. These advances alter the criteria (panels of biomarkers) used to classify individuals and disease subtypes and indicate that new biomarkers will need to be identified for each modification of therapy.

## Supporting Information

Figure S1 ROC curves for the individual genes in the signature (MA analysis). Genes that are induced in the NR state are found above the diagonal whereas those that are repressed in the NR state are found below the diagonal. AUC is the area under the curve.
(PDF)

Figure S2 Summary ROC curves for the gene signature. ROC curves are shown for the MA and TLDA analyses for all 10 genes of the signature incorporating or not the HPV status using the weighted gene sums from the Support Vector Machines supervised prediction method. AUC is the area under the curve.
(PDF)

Figure S3 An interactome network, as visualized by Cytoscape. Associations are depicted among the protein products of the 10-gene classifier and other proteins. The red and green colors indicate the up- and down-regulated genes, respectively, in the 10-gene classifier and the yellow colors are the proteins that associate with members of the classifier. The edges connect proteins for which experimental evidence of physical interaction has been reported in several databases. The lengths and thicknesses of the edges are arbitrary. All of the protein members of the signature (except ODZ2) are linked to another member by one or at most by two interactor proteins. The vast majority of the interactor proteins (24/27) are involved in the regulation of gene expression via chromatin remodeling, signal transduction and RNA metabolism with the others (3/27) involved in protein synthesis and folding (Table S2). Many of these interactions occur in the context of multi-protein complexes, the cellular functions of which are wide ranging and not completely deciphered. The “proximity” of the members of the 10 gene classifier in the interactome network is intriguing and, perhaps, an indication of the specificity of the classifier.
(PDF)

Table S1 **TLDA Gene Expression Ratios**
(PDF)

Table S2 **GO Functions of Proteins Associating with Members of the Gene Signature.**
(PDF)

## References

[1] Braakhuis BJ, Brakenhoff RH, Leemans CR (2005) Second field tumors: a new opportunity for cancer prevention? Oncologist 10: 493–500. 16079316 10.1634/theoncologist.10-7-493

[2] Chen AY, Schrag N, Hao Y, et al (2007) Changes in treatment of advanced oropharyngeal cancer, 1985–2001. Laryngoscope 117: 16–21. 17202924 10.1097/01.mlg.0000240182.61922.31

[3] The Department of Veteran Affairs Laryngeal Cancer Study Group (1991) Induction chemotherapy plus radiation compared with surgery plus radiation in patients with advanced laryngeal cancer. N Engl J Med 324: 1685–1690. 2034244 10.1056/NEJM199106133242402

[4] Forastiere AA, Goepfert H, Maor M, Pajak TF, Weber R, et al (2003) Concurrent chemotherapy and radiotherapy for organ preservation in advanced laryngeal cancer. N Engl J Med 349: 2091–2098. 14645636 10.1056/NEJMoa031317

[5] Pignon JP, Bourhis J, Domenge C, Designé L (2000) Chemotherapy added to locoregional treatment for head and neck squamous cell carcinoma: three meta-analyses of updated individual data. MACH-NC Collaborative Group. Meta-analysis chemotherapy on head and neck cancer. Lancet 355: 949–955. 10768432

[6] Cmelak AJ, Li S, Goldwasser MA, Murphy B, Cannon M, et al (2007) Phase II trial of chemoradiation for organ preservation in respectable stage III or IV squamous cell carcinomas of the larynx or oropharynx: results of Eastern Cooperative Oncology Group Study E2399. J Clin Oncol 25: 3971–3977. 17761982 10.1200/JCO.2007.10.8951

[7] Posner MR, Hershock DM, Blajman CR, Mickiewicz E, Winquist E, et al (2007) TAX 324 Study Group. Cisplatin and fluorouracil alone or with docetaxel in head and neck cancer. N Engl J Med 357: 1705–1715. 17960013 10.1056/NEJMoa070956

[8] Vermorken JB, Remenar E, van Herpen C, Gorlia T, Mesia R, et al (2007) Cisplatin, fluorouracil, and docetaxel in unresectable head and neck cancer. N Engl J Med 357: 1695–1704. 17960012 10.1056/NEJMoa071028

[9] Lorch JH, Goloubeva O, Haddad RI, Cullen K, Sarlis N, et al (2011) Induction chemotherapy with cisplatin and fluorouracil alone or in combination with docetaxel in locally advanced squamous-cell cancer of the head and neck: long-term results of the TAX 324 randomised phase 3 trial. Lancet Oncol 12: 153–159. 21233014 10.1016/S1470-2045(10)70279-5 PMC4356902

[10] Blons H, Gad S, Zinzindohoue F, Manière I, Beauregard J, et al (2004) Matrix metalloproteinase 3 polymorphism: a predictive factor of response to neoadjuvant chemotherapy in head and neck squamous cell carcinoma. Clin Cancer Res 10: 2594–2599. 15102660 10.1158/1078-0432.ccr-1116-03

[11] Rodriguez-Pinilla M, Rodriguez-Peralto JL, Hitt R, Sanchez JJ, Ballestin C, et al (2004) Cyclin A as a predictive factor for chemotherapy response in advanced head and neck cancer. Clin Cancer Res 10: 8486–8492. 15623629 10.1158/1078-0432.CCR-04-0771

[12] Nemeth Z, Szigeti K, Mathe M, Szabó G, Velich N, et al (2005) Effect of induction chemotherapy on changes of laminin and syndecan expression in oral squamous cell carcinomas: a prospective, randomized, clinicopathologic and immunohistochemical study. J Craniofac Surg 16: 205–212. 15750416 10.1097/00001665-200503000-00005

[13] Chung CH, Parker JS, Karaca G, Wu J, Funkhouser WK, et al (2004) Molecular classification of head and neck squamous cell carcinomas using patterns of gene expression. Cancer Cell 5: 489–500. 15144956 10.1016/s1535-6108(04)00112-6

[14] Rickman DS, Millon R, De Reynies A, Thomas E, Wasylyk C, et al (2008) Prediction of future metastasis and molecular characterization of head and neck squamous-cell carcinoma based on transcriptome and genome analysis by microarrays. Oncogene 27: 6607–6622. 18679425 10.1038/onc.2008.251

[15] Colombo J, Fachel AA, De Freitas Calmon M, Cury PM, Fukuyama EE, et al (2009) Gene expression profiling reveals molecular marker candidates of laryngeal squamous cell carcinoma. Oncol Rep 21: 649–663. 19212623

[16] Luthra R, Wu TT, Luthra MG, Izzo J, Lopez-Alvarez E, et al (2006) Gene expression profiling of localized esophageal carcinomas: association with pathologic response to preoperative chemoradiation. J Clin Oncol 24: 259–267. 16344314 10.1200/JCO.2005.03.3688

[17] Motoori M, Takemasa I, Yamasaki M, Komori T, Takeno A, et al (2010) Prediction of the response to chemotherapy in advanced esophageal cancer by gene expression profiling of biopsy samples. Int J Oncol 37: 1113–1120. 20878059 10.3892/ijo_00000763

[18] Oken MM, Creech RH, Tormey DC, Horton J, Davis TE, et al (1982) Toxicity and response criteria of the Eastern Cooperative Oncology Group. Am J Clin Oncol 5: 649–655. 7165009

[19] Benjamini Y, Hochberg Y (1995) Controlling the false discovery rate A practical and powerful approach to multiple testing. Journal of the Royal Statistical Society B 57: 289–300.

[20] Marisa L, Ichanté JL, Reymond N, Aggerbeck L, Delacroix H, et al (2007) MAnGO: an interactive R-based tool for two-colour microarray analysis. Bioinformatics 23: 2339–2341. 17586547 10.1093/bioinformatics/btm321

[21] Ramaswamy S, Tamayo P, Rifkin R, Mukherjee S, Yeang CH, et al (2001) Multiclass cancer diagnosis using tumor gene-expression signatures. Proceedings of the National Academy of Sciences USA 98: 15149–15154. 10.1073/pnas.211566398 PMC64998 11742071

[22] Fan R-E, Chen P-H, Lin C-J (2005) Working set selection using second order information for training SVM. Journal of Machine Learning Research 6: 1889–1918.

[23] Simon R, Lam A, Li MC, Nqan M, Menenzes S, et al (2006) Analysis of gene expression data using BRB-Array Tools. Cancer Inform 3: 11–17. PMC2675854 19455231

[24] Simon R, Radmacher MD, Dobbin K, McShane LM (2003) Pitfalls in the analysis of DNA microarray data: Class prediction methods. Journal of the National Cancer Institute 95: 14–18. 12509396 10.1093/jnci/95.1.14

[25] Deleeuw J (1977) In Recent Developments in Statistics. 1. R. Barra, et al. eds. North-Holland. Amsterdam.

[26] Kruskal JB, Wish M, Uslaner EM (2006) Multidimensional scaling. Sage Publications, Beverly Hills, California.

[27] Spiess C, Meyer AS, Reissmann S, Frydman J (2004) Mechanism of the eukaryotic chaperonin: protein folding in the chamber of secrets. Trends Cell Biol 14: 598–604. 15519848 10.1016/j.tcb.2004.09.015 PMC2812437

[28] Grantham J, Brackley KI, Willison KR (2006) Substantial CCT activity is required for cell cycle progression and cytoskeletal organization in mammalian cells. Exp. Cell Res 312: 2309–2324. 10.1016/j.yexcr.2006.03.028 16765944

[29] Coghlin C, Carpenter B, Dundas SR, Lawrie LC, Telfer C, et al (2006) Characterization and over-expression of chaperonin t-complex proteins in colorectal cancer. J Pathol 210: 351–357. 16981251 10.1002/path.2056

[30] Macleod K, Mullen P, Sewell J, Rabiasz G, Lawrie S, et al (2005) Altered ErbB receptor signaling and gene expression in cisplatin-resistant ovarian cancer. Cancer Res 65: 6789–6800. 16061661 10.1158/0008-5472.CAN-04-2684

[31] Yamaguchi H, Condeelis J (2007) Regulation of the actin cytoskeleton in cancer cell migration and invasion. Biochim Biophys Acta 1773: 642–652. 16926057 10.1016/j.bbamcr.2006.07.001 PMC4266238

[32] Stirling PC, Cuéllar J, Alfaro GA, El Khadali F, Beh CT, et al (2006) PhLP3 modulates CCT-mediated actin and tubulin folding via ternary complexes with substrates. J Biol Chem 28: 7012–7021. 10.1074/jbc.M513235200 16415341

[33] Samimi G, Manorek G, Castel R, Breaux JK, Cheng TC, et al (2005) cDNA microarray-based identification of genes and pathways associated with oxaliplatin resistance. Cancer Chemother Pharmacol 55: 1–11. 15378272 10.1007/s00280-004-0819-9

[34] Calderwood SK, Khaleque MA, Sawyer DB, Ciocca DR (2006) Heat shock proteins in cancer: chaperones of tumorigenesis. Trends Biochem Sci 31: 164–172. 16483782 10.1016/j.tibs.2006.01.006

[35] D’Souza B, Miyamoto A, Weinmaster G (2008) The many facets of Notch ligands. Oncogene 27: 5148–5167. 18758484 10.1038/onc.2008.229 PMC2791526

[36] Stansky N, Egloff AM, Tward AD, Kostic AD, Cibulskis K, et al (2011) The mutational landscape of head and neck squamous cell carcinoma. Science 333: 1157–1160. 21798893 10.1126/science.1208130 PMC3415217

[37] Snijders AM, Schmidt BL, Fridlyand J, Dekker N, Pinkel D, et al (2005) Rare amplicons implicate frequent deregulation of cell fate specification pathways in oral squamous cell carcinoma. Oncogene 24: 4232–4242. 15824737 10.1038/sj.onc.1208601

[38] Zaravinos A, Lambrou GI, Boulalas I, Delakas D, Spandidos DA (2011) Identification of common differentially expressed genes in urinary bladder cancer. PLoS ONE 6: e18135. 21483740 10.1371/journal.pone.0018135 PMC3070717

[39] Chen M, Tominaga K, Pereira-Smith OM (2010) Emerging role of the MORF/MRG gene family in various biological processes, including aging. Ann N Y Acad Sci 1197: 134–141. 20536842 10.1111/j.1749-6632.2010.05197.x PMC2918256

[40] Banerjea A, Ahmed S, Hands RE, Huang F, Han X, et al.. (2004) Colorectal cancers with microsatellite instability display mRNA expression signatures characteristic of increased immunogenicity. Mol Cancer 3: 21–. 10.1186/1476-4598-3-21 PMC514528 15298707

[41] Wolin SL, Cedervall T (2002) The La protein. Annu Rev Biochem 71: 375–403. 12045101 10.1146/annurev.biochem.71.090501.150003

[42] Sommer G, Rossa C, Chi AC, Neville BW, Heise T (2011) Implication of RNA-binding protein La in proliferation, migration and invasion of lymph node-metastasized hypopharyngeal SCC cells. PLoS ONE 6: e25402. 22016766 10.1371/journal.pone.0025402 PMC3189910

[43] Yokoyama A, Yamashita T, Shiozawa E, Nagasawa A, Okabe-Kado J, et al (2004) MmTRA1b/phospholipid scramblase 1 gene expression is a new prognostic factor for acute myelogenous leukemia. Leuk Res 28: 149–157. 14654079 10.1016/s0145-2126(03)00189-9

[44] Lu B, Sims PJ, Wiedmer T, Moser AH, Shiqenaqa JK, et al (2007) Expression of the phospholipid scramblase (PLSCR) gene family during the acute phase response. Biochim Biophys Acta 1771: 1177–1185. 17590392 10.1016/j.bbalip.2007.05.002

[45] Silverman RH, Halloum A, Zhou A, Dong B, Al-Zoghaibi F, et al (2002) Suppression of ovarian carcinoma cell growth in vivo by the interferon-inducible plasma membrane protein, phospholipid scramblase 1. Cancer Res 62: 397–402. 11809687

[46] Monteclaro FS, Vogt PK (1993) A Jun-binding protein related to a putative tumor suppressor. Proc Natl Acad Sci U S A. 90: 6726–6730. 10.1073/pnas.90.14.6726 PMC47005 8341691

[47] Workman P, Clarke PA, Al-Lazikani B (2012) Personalized Medicine: Patient-Predictive Panel Power. Cancer Cell 21: 455–456. 22516256 10.1016/j.ccr.2012.03.030

[48] Garnett MJ, Edelman EJ, Heidorn SJ, Greenman CD, Dastur A, et al (2012) Systematic identification of genomic markers of drug sensitivity in cancer cells. Nature 483 570–575. 22460902 10.1038/nature11005 PMC3349233

[49] Barretina J, Caponigro G, Stransky N, Venkatesan K, Margolin AA, et al (2012) The Cancer Cell Line Encyclopedia enables predictive modelling of anticancer drug sensitivity. Nature 483: 603–607. 22460905 10.1038/nature11003 PMC3320027

