# Supplementary figures and images for: A Head and Neck Cancer Tumor Response-Specific Gene Signature for Cisplatin, 5-Fluorouracil Induction Chemotherapy Fails with Added Taxanes
Source: PLoS One. 2012 Oct 9;7(10):e47170. doi: 10.1371/journal.pone.0047170 (PMC3467249; doi:10.1371/journal.pone.0047170)

ROC Curves for Individual Genes in the Signature (MA Analysis)

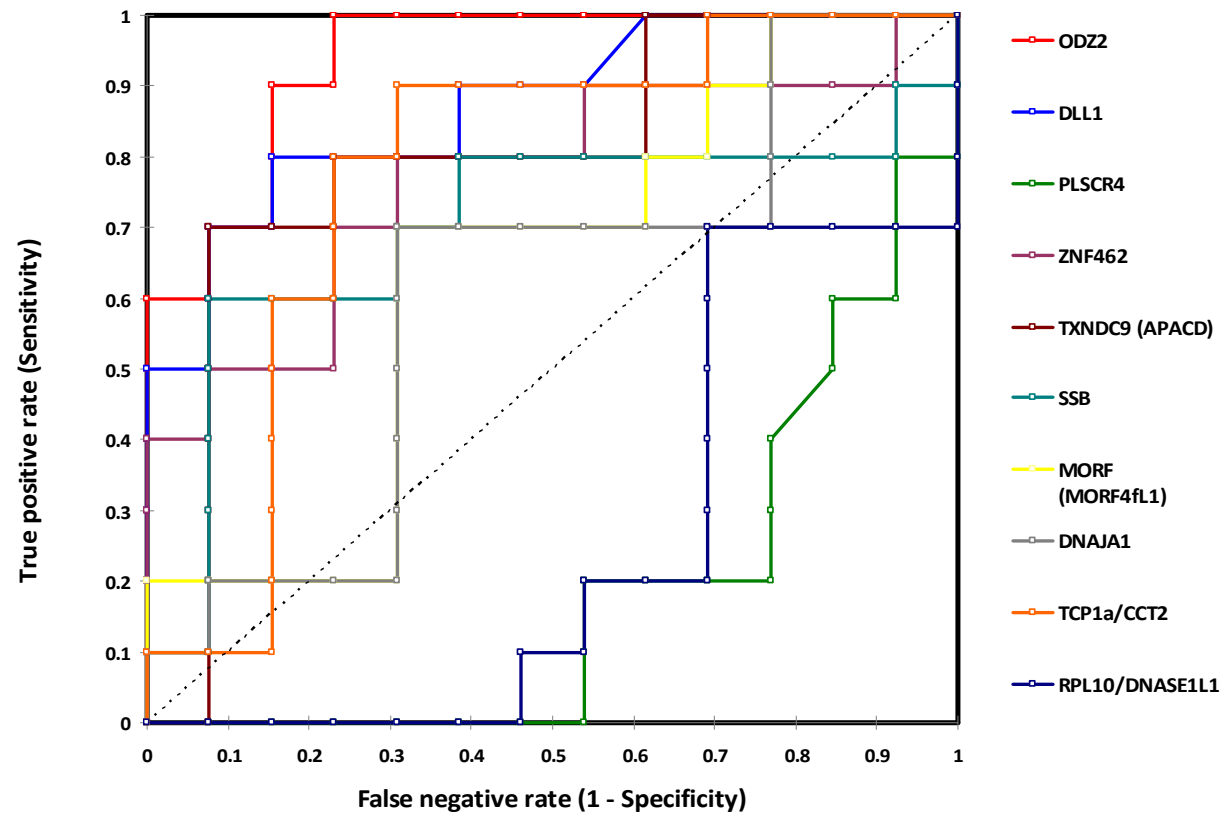

Supplement: Figure S1 — ROC curves for the individual genes in the signature (MA analysis). Genes that are induced in the NR state are found above the diagonal whereas those that are repressed in the NR state are found below the diagonal. AUC is the area under the curve. (PDF) [file pone.0047170.s001.pdf]

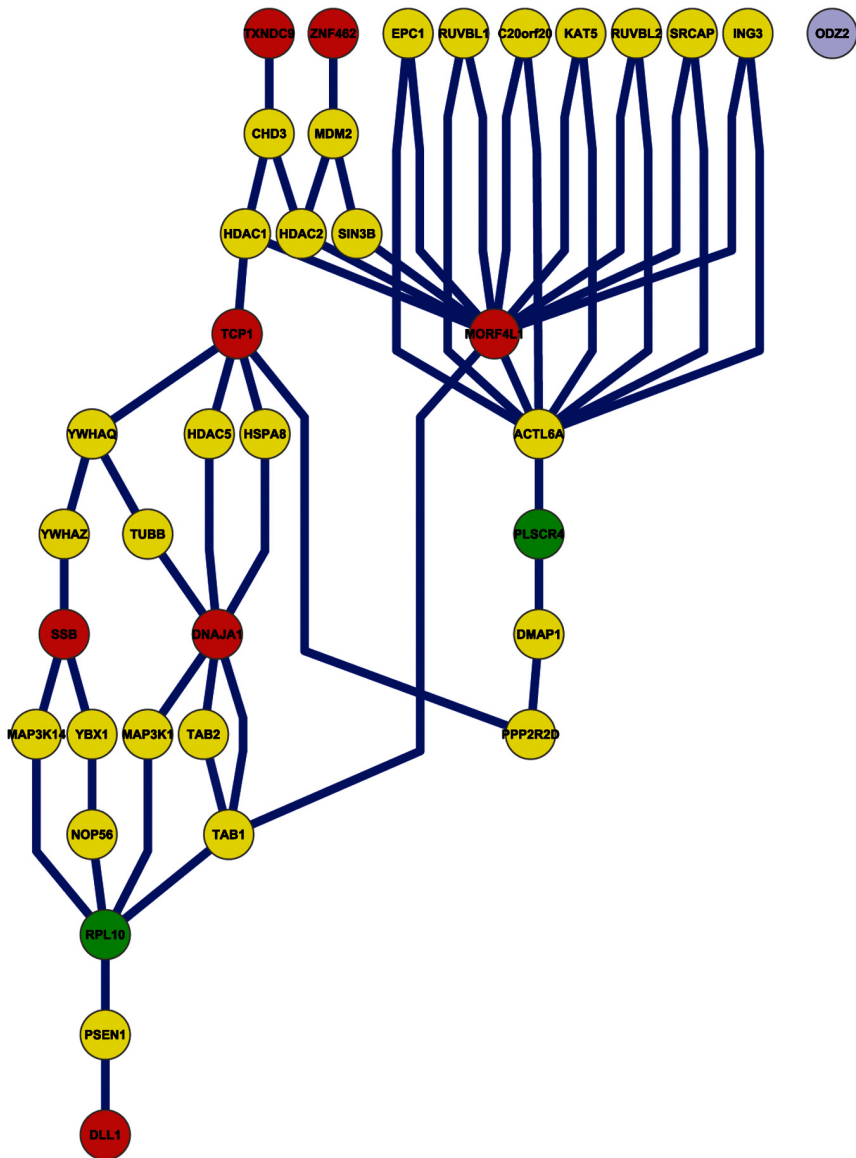

Supplement: Figure S3 — An interactome network, as visualized by Cytoscape. Associations are depicted among the protein products of the 10-gene classifier and other proteins. The red and green colors indicate the up- and down-regulated genes, respectively, in the 10-gene classifier and the yellow colors are the proteins that associate with members of the classifier. The edges connect proteins for which experimental evidence of physical interaction has been reported in several databases. The lengths and thicknesses of the edges are arbitrary. All of the protein members of the signature (except ODZ2) are linked to another member by one or at most by two interactor proteins. The vast majority of the interactor proteins (24/27) are involved in the regulation of gene expression via chromatin remodeling, signal transduction and RNA metabolism with the others (3/27) involved in protein synthesis and folding (Table S2). Many of these interactions occur in the context of multi-protein complexes, the cellular functions of which are wide ranging and not completely deciphered. The “proximity” of the members of the 10 gene classifier in the interactome network is intriguing and, perhaps, an indication of the specificity of the classifier. (PDF) [file pone.0047170.s003.pdf]
