# Supplementary material for: A Head and Neck Cancer Tumor Response-Specific Gene Signature for Cisplatin, 5-Fluorouracil Induction Chemotherapy Fails with Added Taxanes
Source: PLoS One. 2012 Oct 9;7(10):e47170. doi: 10.1371/journal.pone.0047170 (PMC3467249; doi:10.1371/journal.pone.0047170)
Supplement: Figure S2 — Summary ROC curves for the gene signature. ROC curves are shown for the MA and TLDA analyses for all 10 genes of the signature incorporating or not the HPV status using the weighted gene sums from the Support Vector Machines supervised prediction method. AUC is the area under the curve. (PDF) [file pone.0047170.s002.pdf]

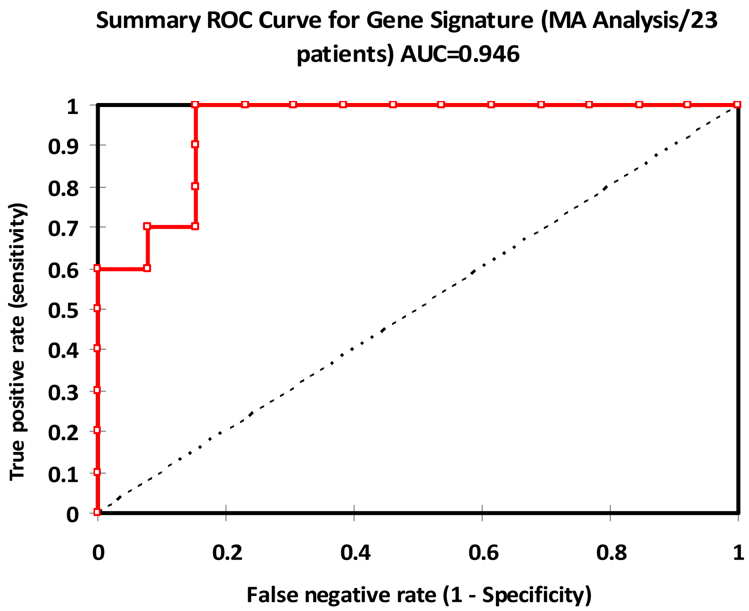
Summary ROC Curve for Gene Signature (MA Analysis/23 patients) AUC=0.946
True positive rate (sensitivity)
False negative rate (1 - Specificity)
0
0.1
0.2
0.3
0.4
0.5
0.6
0.7
0.8
0.9
1
0
0.2
0.4
0.6
0.8
1

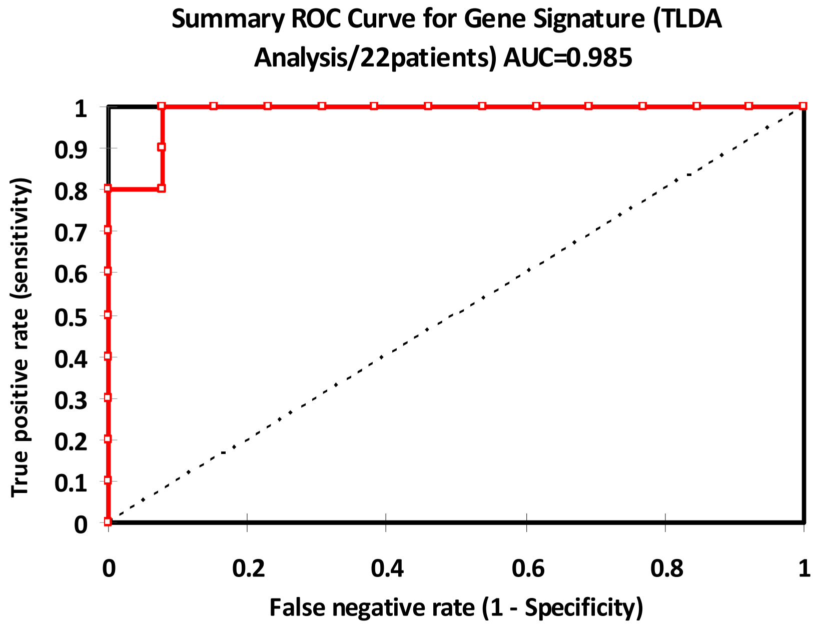
Summary ROC Curve for Gene Signature (TLDA Analysis/22patients) AUC=0.985
True positive rate (sensitivity)
False negative rate (1 - Specificity)
0
0.1
0.2
0.3
0.4
0.5
0.6
0.7
0.8
0.9
1
0
0.2
0.4
0.6
0.8
1

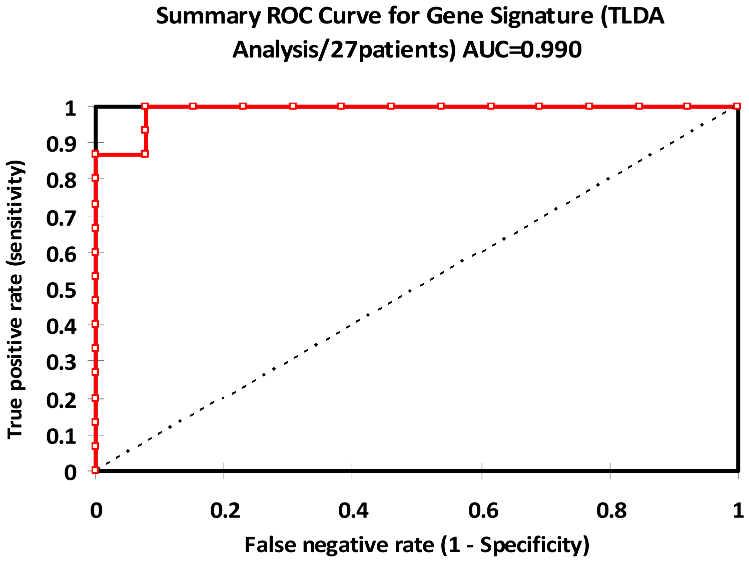
Summary ROC Curve for Gene Signature (TLDA Analysis/27patients) AUC=0.990
True positive rate (sensitivity)
False negative rate (1 - Specificity)
0
0.1
0.2
0.3
0.4
0.5
0.6
0.7
0.8
0.9
1
0
0.2
0.4
0.6
0.8
1

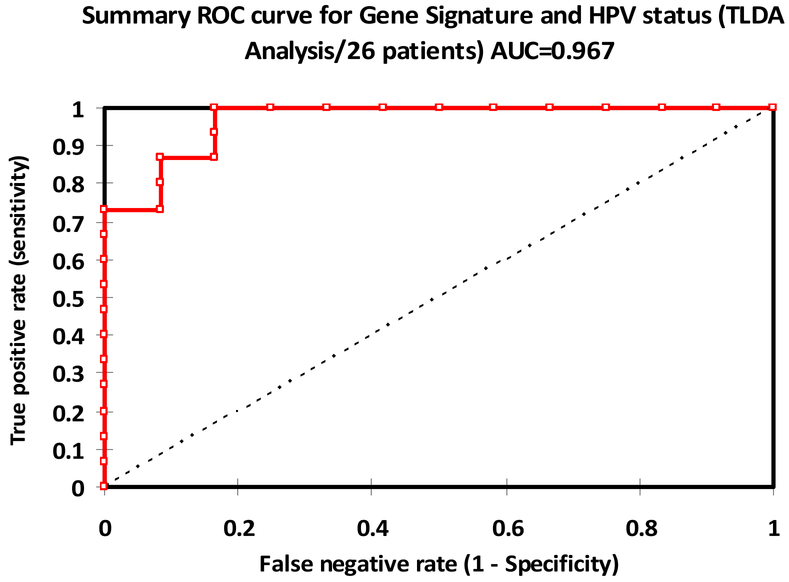
Summary ROC curve for Gene Signature and HPV status (TLDA Analysis/26 patients) AUC=0.967
True positive rate (sensitivity)
False negative rate (1 - Specificity)
0
0.1
0.2
0.3
0.4
0.5
0.6
0.7
0.8
0.9
1
0
0.2
0.4
0.6
0.8
1
