# Supplementary material for: A Head and Neck Cancer Tumor Response-Specific Gene Signature for Cisplatin, 5-Fluorouracil Induction Chemotherapy Fails with Added Taxanes
Source: PLoS One. 2012 Oct 9;7(10):e47170. doi: 10.1371/journal.pone.0047170 (PMC3467249; doi:10.1371/journal.pone.0047170)
Supplement: Table S1 — TLDA Gene Expression Ratios (PDF) [file pone.0047170.s004.pdf]

Supplementary Table 1. TLDA Gene Expression Ratios

Treatment	PF	PF	PF	PF	PF	PF	PF	PF	PF
Gene Name	CCR_Biopsy 40	CCR_Biopsy 57	CCR_Biopsy 109	CCR_Biopsy 110	CCR_Biopsy 121	CCR_Biopsy 130	CCR_Biopsy 156	CCR_Biopsy 157	CCR_Biopsy 164
ODZ2	0.007	0.796	-3.556	-2.989	-2.653	-4.573	0.632	-0.486	1.181
DDL1	-2.644	-1.181	-0.062	-1.718	-1.626	-2.523	-0.042	0.247	0.257
PLSCR4	-1.639	-0.252	-0.201	-0.537	-3.000	-1.450	-2.608	-1.635	1.098
ZNF462	-1.466	-1.751	-0.010	-1.139	-1.742	-1.699	-1.510	-0.876	0.644
TCP1	-1.404	-0.308	-0.431	-0.504	-0.006	-0.168	-0.902	-0.364	0.103
TXNDC9	1.769	0.403	1.092	0.317	0.190	0.566	0.309	0.528	1.707
RPL10	-0.550	0.752	1.642	1.203	1.003	0.589	-0.302	0.862	1.415
SSB	1.009	1.610	1.811	1.289	1.439	1.510	1.356	1.659	2.408
MORF4L1	-5.573	-3.556	-2.548	-2.868	-4.878	-3.381	-4.238	-3.556	-2.265
DNAJA1	-1.446	-1.255	-0.086	-0.913	-1.415	-0.826	-1.404	-0.327	0.399

Treatment	PF	PF	PF	PF	PF	PF	PF	PF	PF
Gene Name	CCR_Biopsy 169	CCR_Biopsy 195	CCR_Biopsy 219_1	CCR_Biopsy 219_2	NR_Biopsy 48	NR_Biopsy 140	NR_Biopsy 143	NR_Biopsy 159	NR_Biopsy 198
ODZ2	0.853	-4.540	1.538	0.174	1.952	1.669	1.980	2.616	2.501
DDL1	0.290	-1.053	0.445	-2.617	0.147	0.908	0.062	0.450	-0.563
PLSCR4	-1.552	-1.502	-1.252	-4.035	-2.806	-2.258	-4.059	-3.556	-3.857
ZNF462	-0.313	-1.811	-0.527	-2.635	-1.396	0.256	-1.165	-0.919	-1.617
TCP1	-0.240	-0.494	-0.178	-1.911	-0.219	0.051	-0.464	-0.687	-0.234
TXNDC9	1.141	0.356	0.805	-0.561	1.236	1.428	1.009	-0.019	0.472
RPL10	0.701	0.993	1.286	-1.114	-0.517	1.851	-1.102	-0.963	-0.742
SSB	2.268	1.391	2.305	0.337	1.685	2.139	1.343	1.667	1.508
MORF4L1	-3.573	-3.158	-3.857	-4.837	-4.293	-2.083	-4.966	-5.108	-3.989
DNAJA1	-0.521	-1.102	-0.333	-2.747	-0.671	0.294	-1.162	-1.304	-1.423

Supplementary Table 1. TLDA Gene Expression Ratios

Treatment	PF	PF	PF	PF	PF	PF	PF	PF	PF
Gene Name	NR_Biopsy 210	NR_Biopsy 223	NR_Biopsy 246	NR_Biopsy 302	NR_Biopsy 303	NR_Biopsy 27	NR_Biopsy 39	NR_Biopsy 76	NR_Biopsy 318
ODZ2	-0.715	-1.120	2.267	2.286	2.038	1.083	2.191	2.663	2.974
DDL1	0.972	0.448	0.134	0.860	1.238	-2.653	-1.366	3.445	0.075
PLSCR4	-2.490	-2.943	-2.442	-4.012	-2.766	-5.878	-3.366	-0.262	-1.388
ZNF462	-0.462	0.385	-0.708	-1.446	0.367	-2.943	-2.608	2.070	-0.233
TCP1	-1.311	-0.184	-0.333	-0.667	-0.293	-1.653	0.107	3.111	-0.228
TXNDC9	0.604	0.641	1.010	0.353	0.754	-0.407	4.016	1.912	0.878
RPL10	-0.111	1.804	0.633	-0.946	0.665	-1.837	-1.474	8.464	0.636
SSB	1.529	1.915	1.409	1.092	2.274	0.102	-0.240	-6.265	2.067
MORF4L1	-4.608	-3.556	-3.989	-5.158	-3.322	-5.442	-6.796	2.016	-5.211
DNAJA1	-0.683	-1.318	-0.844	-1.531	-1.171	-1.595	-0.494	0.800	-0.886

Treatment	PF	T1PF	T1PF	T1PF	T1PF	T1PF	T1PF	T1PF	T1PF
Gene Name	NR_Biopsy 329	CCR_Biopsy 310	CCR_Biopsy 323	CCR_Biopsy 353	CCR_Biopsy 374	NR_Biopsy 138	NR_Biopsy 162_1	NR_Biopsy 162_2	NR_Biopsy 174
ODZ2	0.466	1.176	-2.490	2.308	1.223	0.908	1.524	0.786	1.388
DDL1	0.884	0.045	-2.523	-0.438	-0.556	-1.344	-0.456	-1.130	-0.218
PLSCR4	-2.442	-2.955	-5.796	-3.837	-3.322	-2.171	-3.293	-4.411	-2.366
ZNF462	1.193	-0.771	-4.108	-1.531	-0.955	-1.065	-0.203	-1.454	0.345
TCP1	-0.751	-0.229	-2.482	-0.913	-0.749	-0.214	-0.417	-0.500	-0.152
TXNDC9	0.875	1.155	-0.095	-0.006	0.282	0.881	0.898	0.089	1.000
RPL10	0.876	0.446	-2.699	-0.219	-1.086	0.329	1.396	1.085	1.193
SSB	0.809	0.864	-1.653	1.202	1.559	0.362	0.999	1.737	1.943
MORF4L1	-3.699	-4.035	-5.012	-3.837	-3.900	-3.966	-4.474	-4.796	-2.708
DNAJA1	-1.047	-1.221	-2.474	-0.924	-1.411	-0.983	-0.798	-1.671	-0.653

Supplementary Table 1. TLDA Gene Expression Ratios

Treatment	T1PF	T2PF	T2PF	T2PF	T2PF	T2PF	T2PF	T2PF	T2PF
Gene Name	NR_Biopsy 350	CCR_Biopsy 387	CCR_Biopsy 395	CCR_Biopsy 413	CCR_Biopsy 416	CCR_Biopsy 417	CCR_Biopsy 419	NR_Biopsy 213	NR_Biopsy 427
ODZ2	-0.783	0.898	-2.727	0.847	0.148	1.580	2.580	-0.440	1.848
DDL1	-1.427	0.707	-1.165	-2.388	0.577	-0.170	-0.146	-0.916	-1.842
PLSCR4	-2.796	-1.582	-2.868	-3.756	-1.811	-2.366	-3.035	-2.041	-1.911
ZNF462	-0.798	0.529	-0.935	-2.336	0.324	-0.535	-1.265	-1.188	-1.123
TCP1	-0.884	0.171	-0.701	-1.949	0.302	-0.468	-0.943	0.225	-0.897
TXNDC9	0.163	1.586	0.629	0.377	1.521	1.010	0.141	1.543	0.986
RPL10	-0.595	1.207	0.497	-0.377	2.261	0.418	0.193	0.849	-0.687
SSB	1.659	1.454	1.254	-0.357	2.416	1.096	1.899	1.987	1.126
MORF4L1	-4.756	-3.023	-3.171	-4.035	-3.108	-3.989	-3.626	-4.035	-4.506
DNAJA1	-0.788	-0.149	-0.580	-1.732	-0.102	-0.727	-0.241	-0.868	-1.111

There was insufficient material from CCR_Biopsy_23 for analysis by TLDA; PF=Cisplatin/5FU; T1PF=Cisplatin/5FU/Placitaxel; T2PF=Cisplatin/5FU/Docetaxel

The values correspond to the normalized log_2 ratios sample/reference
