# Supplementary material for: A Head and Neck Cancer Tumor Response-Specific Gene Signature for Cisplatin, 5-Fluorouracil Induction Chemotherapy Fails with Added Taxanes
Source: PLoS One. 2012 Oct 9;7(10):e47170. doi: 10.1371/journal.pone.0047170 (PMC3467249; doi:10.1371/journal.pone.0047170)
Supplement: Table S2 — GO Functions of Proteins Associating with Members of the Gene Signature. (PDF) [file pone.0047170.s005.pdf]

Supplementary Table 2. GO Functions of Proteins Associating with Members of the Gene Signature

PROTEIN ID	FULL NAME	GO FUNCTION
**ACTL6A**	Actin-like 6A	ATP binding, chromatin binding, protein binding, transcription coactivator activity
**SRCAP**	Snf2-related CREBBP activator protein	ATP binding, DNA binding, helicase activity, histone acetyltransferase activity, hydrolase activity, nucleotide binding, transcription coactivator activity
**EPC1**	Enhancer of polycomb homolog 1	Contributes to histone acetyltransferase activity
**KAT5**	K(lysine) acetyltransferase 5	Androgen receptor binding, contributes to histone acetyltransferase activity, metal ion binding, protein binding, repressing transcription factor binding, transcription coactivator activity, transferase activity, transferring acyl groups other than amino-acyl groups
**MDM2**	p53 binding protein homolog	Enzyme binding, ligase activity, metal ion binding, p53 binding, protein binding, ubiquitin-protein ligase activity, zinc ion binding
**RUVBL1**	RuvB-like 1	ATP binding, NOT ATPase activity, DNA helicase activity, hydrolase activity, nucleotide binding, protein binding
**RUVBL2**	RuvB-like 2	ATP binding, ATP-dependent DNA helicase activity, contributes to ATPase activity, DNA helicase activity, damaged DNA binding hydrolase activity, identical protein binding, nucleotide binding, protein binding, unfolded protein binding
**CHD3**	Chromodomain helicase DNA binding protein 3	ATP binding, ATP-dependent DNA helicase activity, DNA binding, helicase activity, hydrolase activity, acting on acid anhydrides, in phosphorus-

		containing anhydrides, metal ion binding, nucleotide binding, protein binding, zinc ion binding
**ING3**	Inhibitor of growth family, member 3	Contributes to histone acetyltransferase activity, metal ion binding, methylated histone residue binding, zinc ion binding
**DMAP1**	DNA methyltransferase 1 associated protein 1	DNA binding, RNA polymerase II repressing transcription factor binding, methyltransferase activity, protein binding, transcription corepressor activity, transferase activity
**C20orf20**	Chromosome 20 open reading frame 20	Chromatin modification, regulation of growth, regulation of transcription, DNA-dependent
**HDAC1**	HDAC2 histone deacetylase 1	NAD-dependent histone deacetylase activity (H3-K14 specific), NAD-dependent histone deacetylase activity (H3-K9 specific), NAD-dependent histone deacetylase activity (H4-K16 specific), RNA polymerase II transcription corepressor activity, activating transcription factor binding, core promoter binding, enzyme binding, histone deacetylase activity (H3-K16 specific), histone deacetylase binding, hydrolase activity, protein binding, protein deacetylase activity, sequence-specific DNA binding transcription factor activity, transcription factor binding
**HDAC2**	HDAC2 histone deacetylase 2	NAD-dependent histone deacetylase activity (H3-K14 specific), NAD-dependent histone deacetylase activity (H3-K9 specific), NAD-dependent histone deacetylase activity (H4-K16 specific), chromatin binding, enzyme binding, histone deacetylase activity, histone deacetylase activity (H3-K16

		specific), hydrolase activity, protein binding, protein deacetylase activity, sequence-specific DNA binding, transcription factor binding
**HDAC5**	HDAC2 histone deacetylase 5	NAD-dependent histone deacetylase activity (H3-K14 specific), NAD-dependent histone deacetylase activity (H3-K9 specific), NAD-dependent histone deacetylase activity (H4-K16 specific), histone deacetylase activity, histone deacetylase activity (H3-K16 specific), hydrolase activity, protein binding, protein kinase C binding, repressing transcription factor binding, transcription factor binding, contributes to transcription regulatory region DNA binding
**YBX1**	Y box binding protein 1	DNA binding, RNA binding, double-stranded DNA binding, protein binding, sequence-specific DNA binding transcription factor activity, single-stranded DNA binding
**PPP2R2D**	Protein phosphatase 2, regulatory subunit B, delta	Phosphatidate phosphatase activity, protein phosphatase type 2A regulator activity
**PSEN1**	Presenilin 1	PDZ domain binding, aspartic-type endopeptidase activity, beta-catenin binding, cadherin binding, calcium channel activity, endopeptidase activity, peptidase activity, protein binding
**YWHAZ**	Tyrosine 3-monooxygenase/tryptophan 5-monooxygenase activation protein, zeta polypeptide	Protein binding, protein domain specific binding, transcription factor binding

**YWHAQ**	Tyrosine 3-monooxygenase/tryptophan 5-monooxygenase activation protein, theta polypeptide	Protein N-terminus binding, protein binding, protein domain specific binding
**MAP3K1**	Mitogen-activated protein kinase kinase kinase 1	ATP binding, JUN kinase binding, JUN kinase kinase kinase activity, MAP kinase kinase kinase activity, MAP/ERK kinase kinase activity, identical protein binding, metal ion binding, mitogen-activated protein kinase binding, mitogen-activated protein kinase kinase binding, nucleotide binding, protein binding, protein kinase activity, protein kinase activity, protein kinase binding, protein serine/threonine kinase activity, sphingolipid binding, ubiquitin-protein ligase activity, zinc ion binding
**MAP3K14**	Mitogen-activated protein kinase kinase kinase 14	ATP binding, MAP kinase kinase kinase activity, NF-kappaB-inducing kinase activity, nucleotide binding, protein binding, protein kinase activity, protein serine/threonine kinase activity
**TAB1**	TGF-beta activated kinase 1/MAP3K7 binding protein 1	Catalytic activity, enzyme activator activity, kinase activator activity, protein binding
**TAB2**	TGF-beta activated kinase 1/MAP3K7 binding protein 2	K63-linked polyubiquitin binding, metal ion binding, protein binding, zinc ion binding
**HSPA8**	Heat shock 70kDa protein 8	ATP binding, ATPase activity, coupled, nucleotide binding, protein binding
**TUBB**	Tubulin, beta class I	GTP binding, GTPase activity, MHC class I protein binding, nucleotide binding, peptide binding, protein complex binding, structural constituent of

		cytoskeleton, structural molecule activity
**NOP56**	Ribonucleoprotein homolog	RNA binding, protein binding, snoRNA binding
**SIN3B**	SIN3 transcription regulator homolog B	Chromatin binding, protein binding, transcription corepressor activity
